# Revealing the Magnetic Structure and Properties of Mn(Co,Ge)_2_

**DOI:** 10.1021/acs.inorgchem.2c02758

**Published:** 2022-10-21

**Authors:** Simon R. Larsen, Vitalii Shtender, Daniel Hedlund, Erna K. Delczeg-Czirjak, Premysl Beran, Johan Cedervall, Alena Vishina, Thomas C. Hansen, Heike C. Herper, Peter Svedlindh, Olle Eriksson, Martin Sahlberg

**Affiliations:** † Department of Chemistry—Ångström, Uppsala University, Box 538, 751 21Uppsala, Sweden; ‡ Department of Materials Science and Engineering, Uppsala University, Box 35, 751 03Uppsala, Sweden; § Department of Physics and Astronomy, Uppsala University, Box 516, SE75120Uppsala, Sweden; ∥ European Spallation Source ESS ERIC, Box 176, 221 00Lund, Sweden; ⊥ Department of Materials and Environmental Chemistry, Stockholm University, 10691Stockholm, Sweden; # Institut Laue-Langevin, 71 Avenue des Martyrs, 38000Grenoble, France; ¶ Nuclear Physics Institute, ASCR, Hlavni 130, 25068Rez, Czech Republic; ∇ School of Science and Technology, Örebro University, SE-701 82Örebro, Sweden

## Abstract

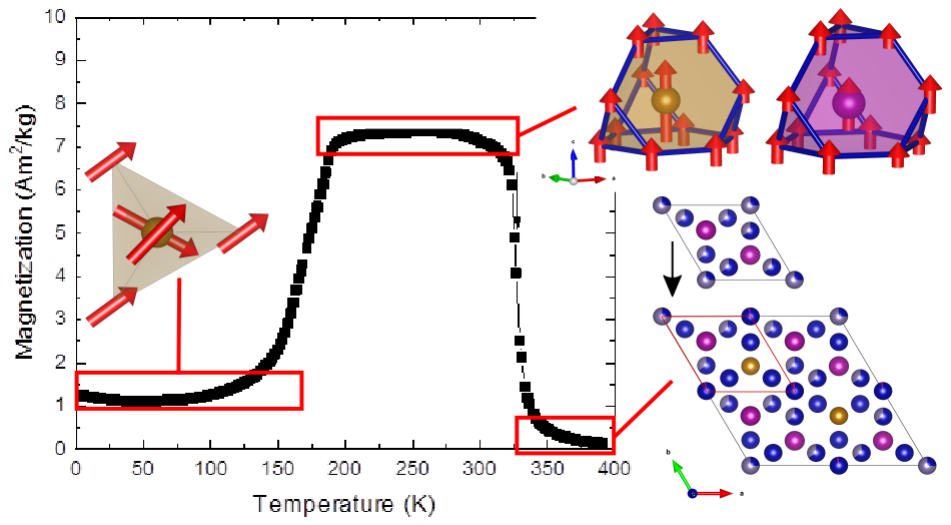

The atomic and magnetic structures of Mn(Co,Ge)_2_ are reported herein. The system crystallizes in the space group *P*6_3_/*mmc* as a superstructure of the MgZn_2_-type structure. The system exhibits two magnetic transitions with associated magnetic structures, a ferromagnetic (FM) structure around room temperature, and an incommensurate structure at lower temperatures. The FM structure, occurring between 193 and 329 K, is found to be a member of the magnetic space group *P*6_3_/*mm*′*c*′. The incommensurate structure found below 193 K is helical with propagation vector **k** = (0 0 0.0483). Crystallographic results are corroborated by magnetic measurements and ab initio calculations.

## Abstract 2

The new candidate material for rare-earth-free permanent magnets, Mn(Co,Ge)_2_, was investigated with neutron diffraction, and its crystalline and magnetic structure was determined. The material exhibits a simple ferromagnetic magnetic structure between 193 and 329 K with individual moments parallel to the *c*-axis. Below 168 K, the moments arrange as a helical structure with magnetic moments revolving around the *c*-axis. Theoretically calculated values agreed well with the model and corroborated the results.

## Introduction

Permanent magnets are an indispensable part of modern society with an ever-increasing importance as they are utilized within the area of renewable energy such as electric vehicles and appliances. Most of the permanent magnets suitable for these applications contain rare earth (RE) elements, which have a large negative impact on the environment when extracted and are rapidly becoming a critical resource.^1^ This has motivated research into finding materials that can replace RE magnets where the superior properties of the RE magnets are less critical, the so-called “gap magnets” which have properties somewhere between those of the RE magnets and the ferrite-based magnets.^2^ By use of data-mining and electronic structure calculations, several RE-free magnetic systems were recently proposed, and one candidate, Mn_2_Co_3_Ge, was successfully synthesized.^3^

The compound Mn_2_Co_3_Ge was first synthesized in 1963^4^ and was reported to crystallize in the space group *P*6_3_/*mmc* with unit cell parameters *a* = 4.803(2) Å and *c* = 7.739(4) Å. It was uncertain whether it assumed a Co–Ge-mixed MgZn_2_-type structure or the ordered Mg_2_Cu_3_Si-type structure. In the recent study of the system,^3^ electronic structure calculations showed that the Mg_2_Cu_3_Si-type structure was energetically favorable, but X-ray diffraction results indicated that the structure was of MgZn_2_ type with *a* = 4.8032(2) Å and *c* = 7.7378(4) Å. It was argued that the Co–Ge intermixing had a detrimental effect on the saturation magnetization, as the ordered structure was predicted to achieve values of 1.71 T, but the Co–Ge-intermixed sample only reached 0.86 T at 10 K. Likewise, it was reasoned that this also reduced the Curie temperature from the originally calculated 700 K to the experimentally obtained 359 K. This Co–Ge intermixing presented also affects how well suited the material is as a permanent magnet. The material was initially reported from calculations to have magnetic hardness parameter,^5^ κ = 0.79, making it a semi-hard magnetic material. Due to the lower saturation magnetization, the material has a higher experimental κ = 1.42. The intermixing was also expected to change the magnetic anisotropy from a uniaxial configuration to an easy-cone; however, at higher temperatures, the uniaxial configuration prevailed. Here, a detailed study of the chemical ordering and the magnetic structure of the system is presented, clarifying previous discrepancies in the literature and explaining the magnetic properties in detail.

## Experimental and Theoretical Methodology

The sample was prepared using high-temperature synthesis methods. Stoichiometric amounts of high-grade Mn (Institute of Physics, Polish Academy of Sciences, 99.995%), Co (Alfa Aesar, 99.9%), and Ge (Kurt J. Lesker, 99.999%) were reacted using induction melting in an Ar atmosphere. The total mass loss during synthesis was less than 2 wt %. The resulting ingot was placed in an Al_2_O_3_ crucible which was in turn sealed in an evacuated quartz glass ampule and annealed at 1073 K for 14 days, followed by water quenching. Preliminary characterization was done using a Bruker D8 X-ray powder diffractometer with a Lynx-eye position-sensitive detector and Cu Kα radiation on a zero-background single-crystal Si sample holder. The microstructure was evaluated with a Zeiss Merlin SEM equipped with a secondary electron detector and an energy-dispersive X-ray spectrometer. The samples for electron microscopy analysis were prepared by standard metallographic techniques through grinding with SiC paper. For the final polishing, a mixture of SiO_2_ and H_2_O was used.

Neutron powder diffraction (NPD) data was collected using the instrument D1b at the Institut Laue-Langevin, Grenoble, France.^6^ The sample was mounted in an 8 mm diameter Vanadium can and placed in an orange cryostat cooled to 1.8 K. Measurements were conducted during heating in a temperature span of 1.8 and 396 K with a wavelength λ = 2.52 Å.^7^ The powder diffraction patterns were analyzed using the Rietveld method^8^ implemented in the FullProf software suite.^9^

Single crystals were collected and subsequently measured using a Bruker D8 single-crystal X-ray diffractometer with Mo Kα radiation (λ = 0.71073 Å). The diffractometer was equipped with an Incoatec Microfocus Source (IμS) and utilized an APEX II CCD area detector. Single-crystal X-ray diffraction (SCXRD) data reduction and numerical absorption corrections were performed using APEX III software from Bruker.^10^ The initial MgZn_2_-based model of the crystal structure was first obtained with the program SHELXT-2014 and refined in the program SHELXL-2014 within the APEX III software package. To differentiate the weak superlattice peaks, CrystalisPro (41.121a) software was used. Structure solution using the Superflip method was carried out using JANA2020, as were model refinements of combined SCXRD and NPD data.

Magnetic measurements were carried out with a Quantum Design MPMS XL system. Field cooled measurements were performed in an applied field of 0.01 T using a cooling rate of 3 K/min. Isothermal magnetization curves were recorded at several temperatures up to 5 T. AC susceptibility versus temperature measurements were performed for three frequencies, 0.17, 1.7, and 170 Hz, with a field amplitude of 0.4 mT. Before performing the AC susceptibility measurements, the ultralow-field option was used to reduce the remanent magnetic field in the setup to below 0.5 μT.

Site and element-resolved magnetic moments were calculated using density functional theory (DFT)^11,12^ formulated within the Lyngby version^13^ of the exact muffin-tin orbital (EMTO) method.^14,15^ The chemical disorder was treated within the coherent potential approximation (CPA).^16,17^ The one-electron Kohn–Sham equations were solved within the soft-core and scalar-relativistic approximations. Furthermore, s, p, d, and f orbitals were included in the basis set. The Green’s function was calculated for 16 complex energy points distributed exponentially on a semi-circular contour including states within 1.1 Ry below the Fermi level. The exchange–correlation effects were described within the local spin density approximation.^18,19^ For the one-center expansion of the full charge density, a  = 8 cutoff was used.  = 0.75 for the on-site screening constant and β_scr_ = 1.1 for the average screening parameter^20,21^ were used to account for the contributions of the screened Coulomb interactions to the one-electron potential of the alloy components. A 12 × 12 × 12 Monkhorst–Pack^22^*k*-mesh grid was used for the Brillouin zone integration. The temperature effect on the local magnetic moments was taken into account considering the thermally induced longitudinal spin fluctuations (LSFs)^13,23,24^ as described in detail in ref^25^. The magnetic disorder at high temperatures was modeled via the disordered local moment (DLM)^26,27^ approach and describes the paramagnetic regime with no net magnetization without accounting for the transversal fluctuations. The structure data from Table 1 was used in a supercell (2 × 2 × 1) to perform the fully relativistic SPRKKR Green’s functions calculation^28,29^ within the local density approximation in the parameterization of Vosko, Wilk, and Nussair.^30,31^ To describe the disorder, we made use of CPA. An 18 × 18 × 18 *k*-mesh was applied for the self-consistent calculation, and the exchange parameters were derived from Liechtenstein’s formula.^32^

**Table 1:** Atomic Positions and Site Occupancy from Model Refinements of Mn(Co_0.78_,Ge_0.22_)_2_ NPD Data^a^

Wyckoff^4^	Split Wyckoff	Atom	*x*	*y*	*z*	Occ.
4*f*	4*f*	Mn	1/3	2/3	0.0599(16)	1
	12*k*	Mn	0.167(1)	0.3335(20)	0.5690(6)	1
2*a*	2*a*	Co	0	0	0	0.2548(3)
	2*a*	Ge	0	0	0	0.7452(3)
	6*g*	Co	1/2	0	0	0.9555(4)
	6*g*	Mn	1/2	0	0	0.0445(4)
6*h*	6*h*(1)	Co	0.5864(16)	0.1729(33)	1/4	1
	6*h*(2)	Co	0.0856(18)	0.1714(36)	1/4	1
	12*j*	Co	0.0850(9)	0.4141(9)	1/4	0.655(1)
	12*j*	Ge	0.0850(9)	0.4141(9)	1/4	0.345(1)

a Uncertainties are given in parentheses.

## Results

### Crystal Structure

**Figure 1:**
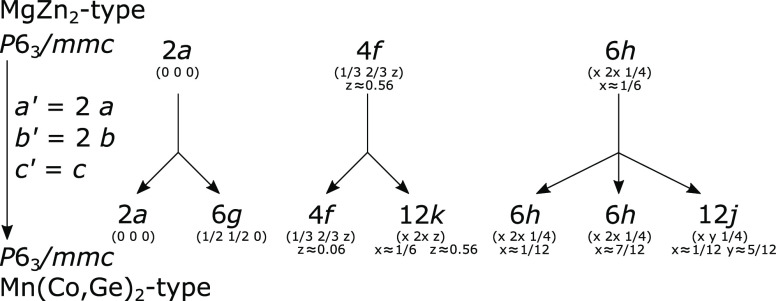
Group–subgroup relations for the MgZn_2_ and Mn(Co,Ge)_2_ structure types showing the site splitting which occurs when the unit cell is doubled in the *a*- and *b*-directions.

In order to investigate the magnetic structure of the system, it is necessary to first establish the atomic structure. Examination of the NPD data collected at 396 K, well into the paramagnetic regime, revealed several peaks that could not be indexed using the unit cell reported previously.^3^ A unit cell with parameters *a* = 9.5932(2) Å and *c* = 7.7440(2) Å using the same space group (*P*6_3_/*mmc*) was able to describe the additional peaks. These values correspond roughly to 2 × *a*, *b* of the MgZn_2_-type unit cell, indicating a superstructure. The expansion of the unit cell causes splitting of the Wyckoff positions, as can be seen in Figure 1.

Having established the presence of a larger unit cell, refinements were set up initially, assuming the fully ordered Mg_2_Cu_3_Si-type structure. Following this, Co and Ge intermixing was introduced stepwise on the 2*a* and 6*h* derived sites. The atomic positions and occupancies extracted from the model are presented in Table 1, and the comparison of the model and data can be found in Figure SI1.

**Figure 2:**
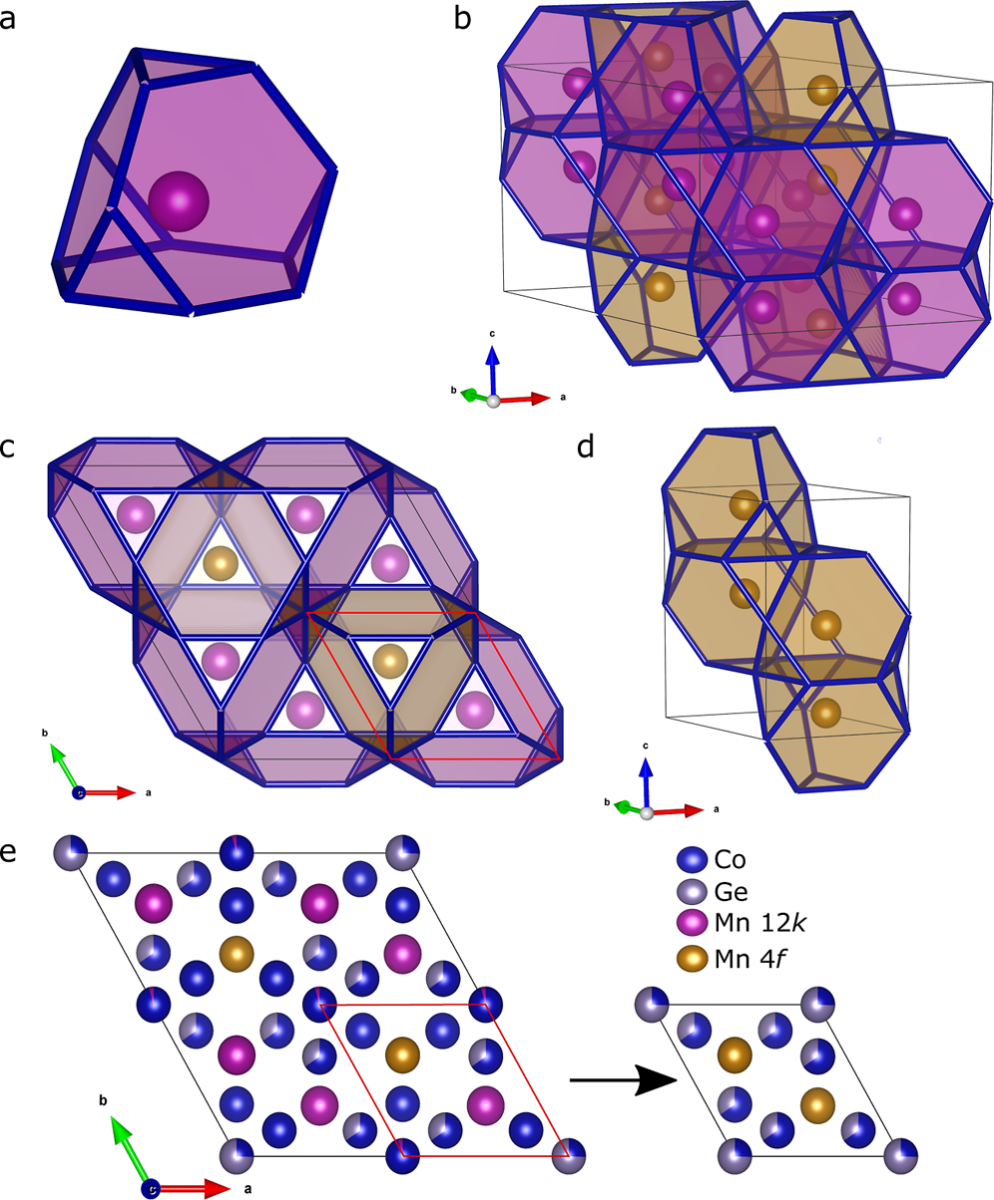
(a) Coordination of Mn by Co and Ge in the Mn(Co_0.78_,Ge_0.22_)_2_ system shown as a Friauf polyhedron. (b) Packing of the polyhedra for the split Mn sites (yellow for 4*f* and purple for 12*k*) in the unit cell. (c) Packing of the polyhedra along the *c*-direction. The red unit cell shows how the two unit cells relate. (d) Polyhedral packing for the small unit cell. (e) Relationship of the occupancy of the unit cell compared to the MgZn_2_-type unit cell.

**Figure 3:**
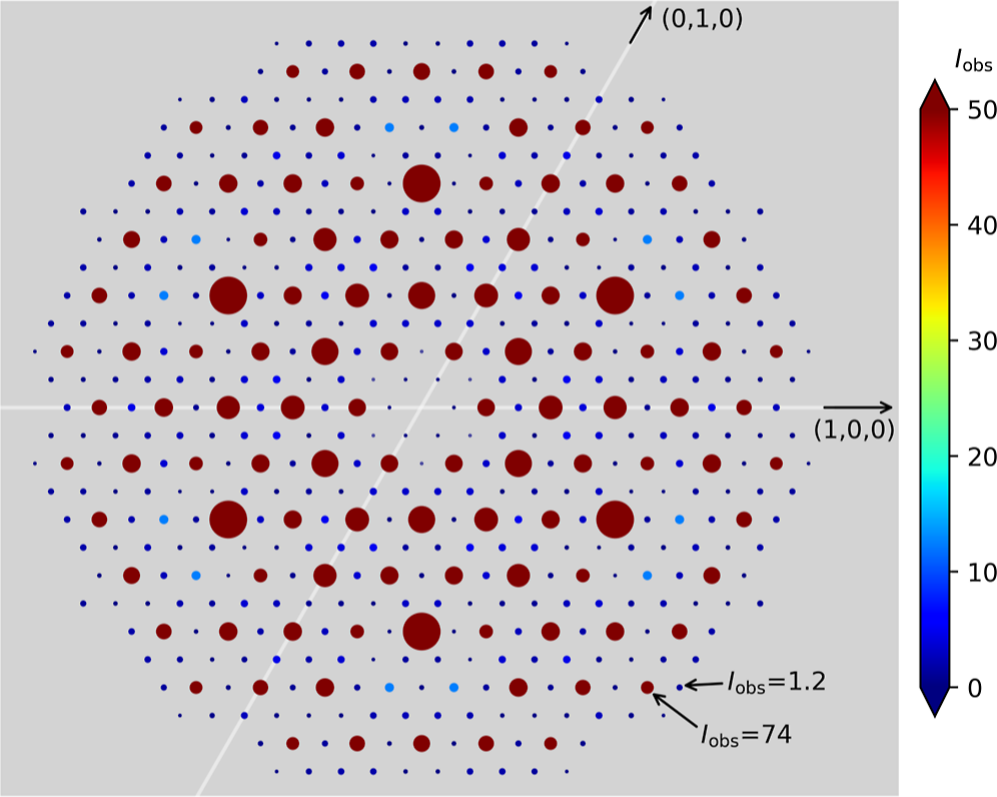
Reflections seen for a single crystal of Mn(Co_0.78_,Ge_0.22_)_2_. The small reflections in blue seen between the main intensities in red are indicative of the larger unit cell.

The structural model is visualized in Figure 2a using face-sharing Friauf polyhedra. The stacking of the two polyhedra, each centered by one of the Mn sites, is shown in Figure 2b–d. In Figure 2e, the relative atomic occupancies are shown for the different atomic sites in the unit cell and compared to the previously reported MgZn_2_-type unit cell. The occupancies varied significantly, with the 2*a* site and the 12*j* site containing most of the Co–Ge intermixing. It should be noted that the 6h and 6g sites likely contain some amount of Ge and Mn in addition to Co. This would account for the difference in stoichiometry (Mn_36_Co_49_Ge_15_ nominally) measured by SEM–energy-dispersive X-ray spectrometry, (Mn_36.7(3)_Co_49.2(3)_Ge_14.1(2)_), and the result of the calculations based on the structure model refinements (Mn_33.9(1)_Co_54.4(1)_Ge_11.7(1)_). This new structure can thus be approximately described as Mn(Co_0.78_,Ge_0.22_)_2_. While there is a more pronounced ordering of the elements on specific sites, it is still imperfect and distinct from Mn_2_Co_3_Ge, and it is given the structure name Mn(Co,Ge)_2_ instead. Examination of SCXRD data presented in Figure 3 revealed subtle reflections, indicating that with good-quality data, the additional ordering can also be seen with X-rays.

### Magnetic Measurements

**Figure 4:**
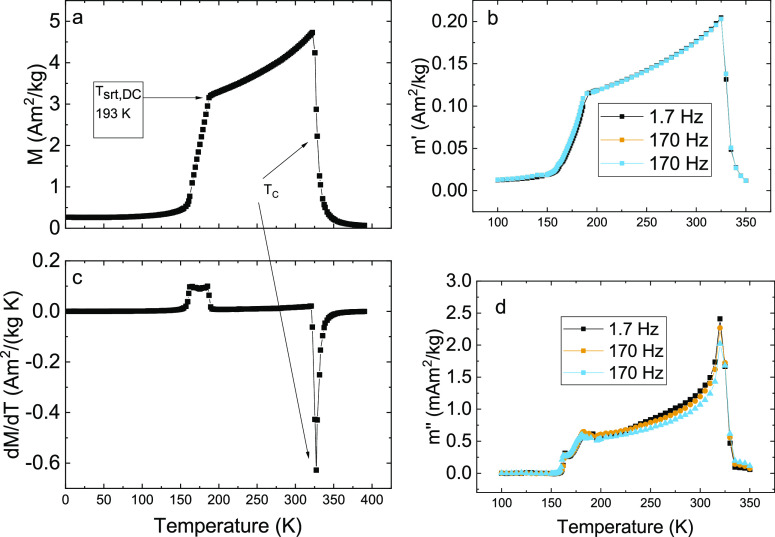
(DC) Magnetization vs temperature of Mn(Co_0.78_,Ge_0.22_)_2_ in an applied field of 0.01 T (a) with corresponding temperature derivative (c). AC magnetization vs temperature of Mn(Co_0.78_,Ge_0.22_)_2_ performed with an AC excitation field of 0.4 mT and frequencies 1.7, 17, and 170 Hz showing the in-phase-component (b) (*m*′) and out-of-phase (d) (*m*″) components of magnetization. For interpretation of the temperature *T*_SRT,DC_, the reader is referred to the main text.

Figure 4 shows the results from temperature-dependent direct current (DC) magnetization and alternating current (AC) magnetization measurements. First, looking at Figure 4a,c, it can be seen that around the temperature of 329 K, the system becomes paramagnetic. In the previous study,^3^ the Curie–Weiss law was used to determine this temperature; however, this system is not well described by the Curie–Weiss approach; therefore, the minimum of d*M*/d*T* was used instead. Here, the difference is only 1 K, but for other compositions (not included in this study), the difference is larger. In the previous study, the sudden decrease in the magnetization versus temperature curve, marked in Figure 4a as *T*_SRT,DC_ = 193 K, was suggested to be the onset of an incommensurate antiferromagnetic (AF) cone structure.^3^

The AC magnetization results shown in Figure 4b,d suggest that this onset of the incommensurate structure occurs at a slightly lower temperature. First, the temperature dependence of the in-phase component of the AC magnetization shown in Figure 4b resembles the temperature dependence of the DC magnetization. The frequency dependence of the in-phase component is weak, but the fact that an out-of-phase component appears below the ferromagnetic (FM) ordering temperature (Figure 4d) proves that a slow magnetic relaxation process exists in the FM state.

The origin of the relaxation process can be seen by considering the energy in a ferro- or ferrimagnetic material. The relevant energies here are the Zeeman energy and the magnetostatic energy, where the latter is the important part. The magnetostatic energy is reduced by the formation of magnetic domains and domain walls, while the Zeeman energy is reduced by moving domain walls to increase the field-induced magnetization. The slow magnetic relaxation revealed by the out-of-phase component of the AC magnetization corresponds to the domain wall motion. Figure 4d shows that the magnetic loss begins to disappear around 180 K, and at *T*_SRT,AC_ = 168 K, the out-of-phase component (*m*″) becomes zero, which is a sign that the incommensurate AF cone structure has been established. Between the temperatures 180 and 168 K, the out-of-phase component exhibits a gradual decrease in magnitude, while the last sharp decrease in the out-of-phase component occurs in a narrow temperature range. This gradual decrease in the out-of-phase component is taken as an indication that there is an intermediate spin structure present between 180 and 168 K.

In the (pure) AF state, there are no magnetic domain walls that can move in response to the alternating magnetic field, and therefore, there will be no losses associated with it in this state. To complement the temperature-dependent measurements, isothermal magnetization curves up to 5 T were performed at various temperatures.

**Figure 5:**
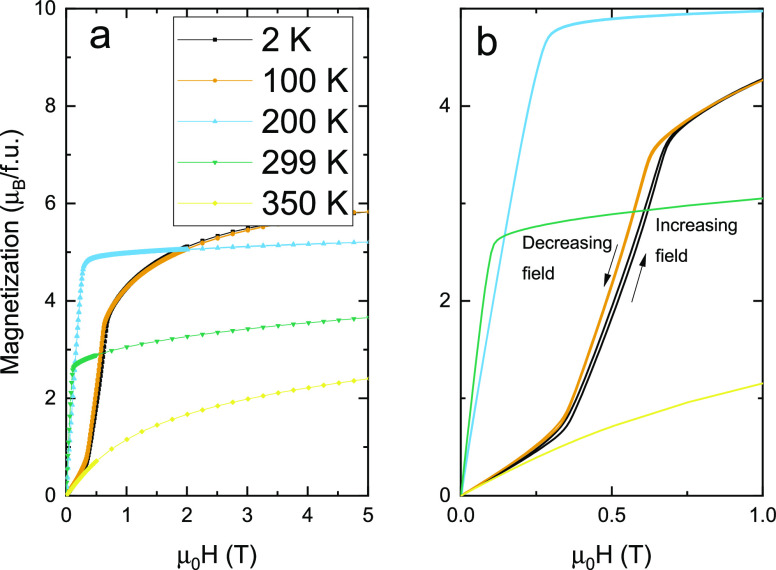
Isothermal magnetization curves of Mn(Co_0.78_,Ge_0.22_)_2_ for temperatures 2, 100, 200, 299, and 350 K shown in the field range 0–5 (a) and 0–1 T in (b).

These are presented in Figure 5a (0–5 T) and in Figure 5b (0–1 T) with the field first being increased from 0 to 5 T and then decreased back to 0 T. The temperatures 299 and 200 K show normal FM behavior with magnetization values reaching 5.34 μ_B_/fu at 200 K and 3.66 μ_B_/fu at 299 K. In the incommensurate AF cone state, the magnetization versus field measurement curves reveal field-driven (metamagnetic) first-order spin-flop transitions. This is best seen in Figure 5b for the magnetization versus field at 2 K (solid black line) but is also visible at 100 K (orange curve). Referring to isothermal magnetization at 2 K, between 0 and 0.34 T, the magnetization versus field curve resembles that of an AF; that is, the response to the magnetic field is weak and linear in field. Above 0.34 T, the magnetization versus field curve steeply increases, which is indicative of a spin-flop (metamagnetic) transition. The isothermal magnetization exhibits magnetic hysteresis; the process is first-order like since the traces back and forth are not the same. These results are consistent with an incommensurate AF order at low temperatures and a field-driven spin-flop transition.

### FM Structure, 193–329 K

**Figure 6:**
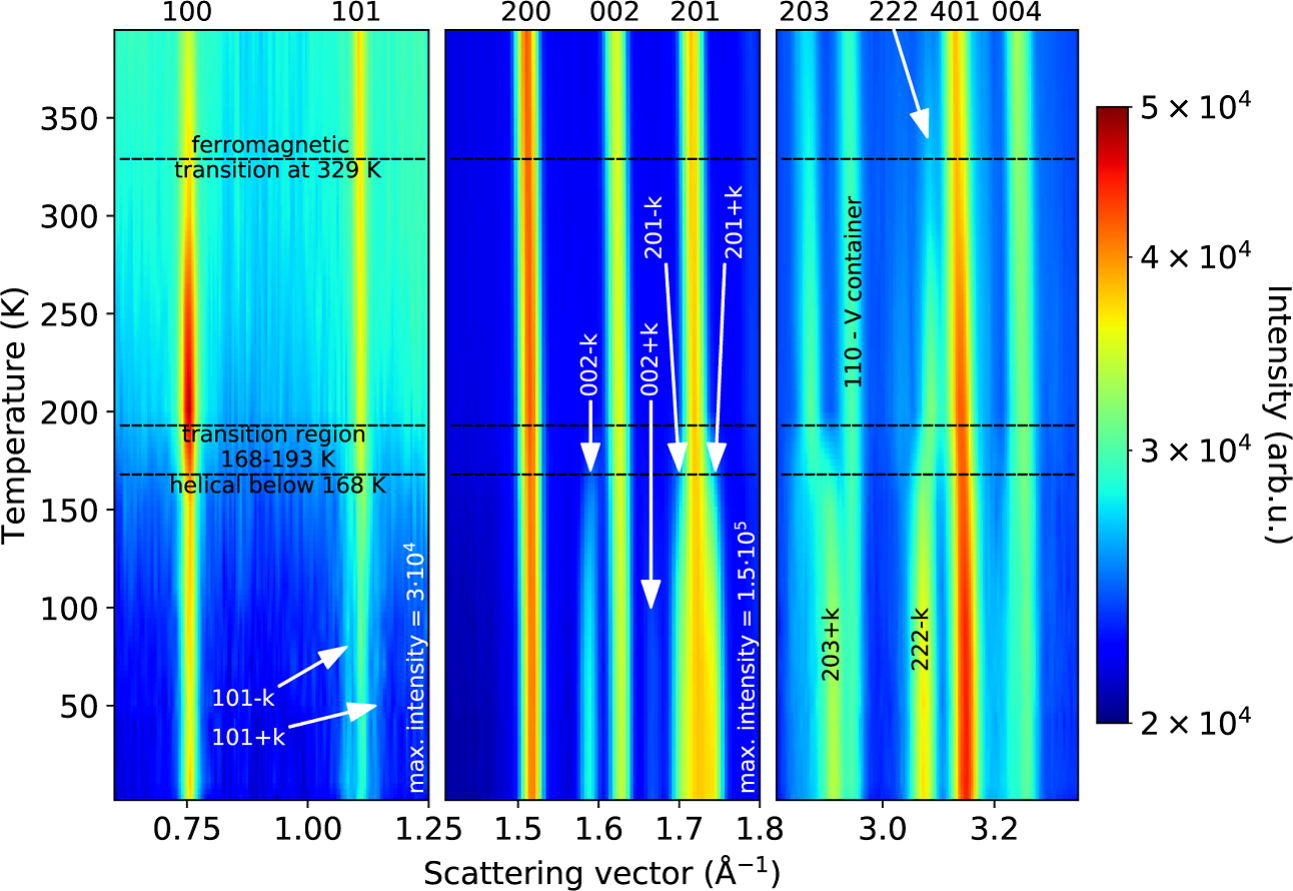
Evolution of the strongest magnetic reflections seen in the neutron diffraction patterns of Mn(Co_0.78_,Ge_0.22_)_2_. The paramagnetic state is seen above 329 K.

Figure 6 shows how the NPD patterns change with temperature. This section will concern the FM configuration spanning the temperature range of 193–329 K. In this region, the increase in intensity coincides with the crystallographic reflections, making the most reasonable propagation vector **k** = (0 0 0). The symmetry analysis performed using the k-SUBGROUPSMAG tool of BCS (Bilbao Crystallographic Server)^33^ and IsoDistort Software Suite^34,35^ pointed to several possible magnetic arrangements. All of them were tested against the measured data. The only model which was able to match the experimental data has the magnetic space group *P*6_3_/*mm*′*c*′ (BNS 194.270) with unit cell parameters *a* = 9.5715(2) Å and *c* = 7.7275(2) Å, leading to a FM arrangement along the *c*-axis.

**Figure 7:**
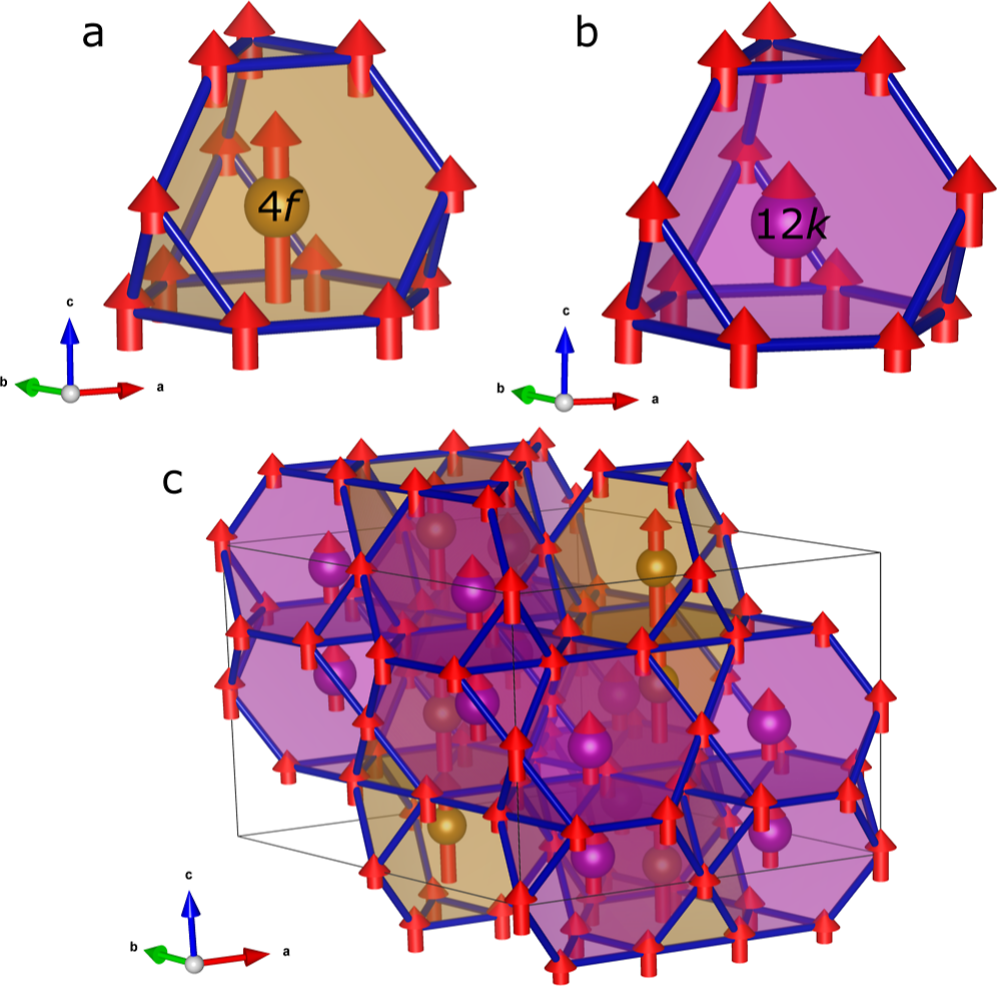
(a) Coordination polyhedron for the 4*f* Mn with magnetic moments of surrounding atoms at 200 K. (b) Coordination polyhedron with magnetic moments for 12*k* Mn. (c) Structure of Mn(Co_0.78_,Ge_0.22_)_2_ built by stacking these polyhedra.

A model was established using the elemental distributions attained from the NPD model in the paramagnetic regime, and similar magnetic moments were assumed for atoms in similar local environments. In this case, the 6*g* and 6*h*(2) sites, which are coordinated by several Ge-rich sites, were kept the same, as were the sites mainly coordinated by Co, 6*h*(1), and 12*j*. The Mn sites were initially assumed to have similar moments, but in all model attempts, a better agreement with the data was achieved when they were refined individually. The resulting model is visualized in Figure 7, while the comparison of model and data can be found in Figure SI2.

The total magnetic moment at 200 K was found to be 6.33 μ_B_/fu, which agrees well with the 5.34 μ_B_/fu measured by magnetometry. The small discrepancy likely relates to the intermixing on the 6*h* sites, which could not be quantified with the available data.

### Incommensurate Structure, *T* < 168 K

**Figure 8:**
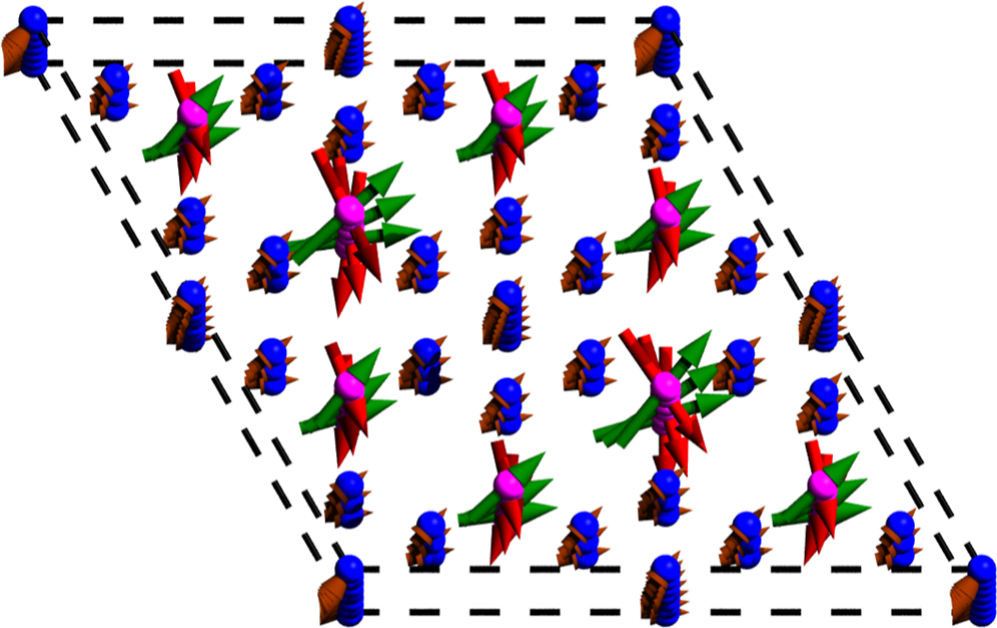
Evolution of the magnetic moments of the incommensurate structure through three unit cells of Mn(Co_0.78_,Ge_0.22_)_2_, seen along the *c*-axis. The green and red arrows are assigned to one of each of the split Mn pairs.

Below 193 K, weak satellite reflections appear near the atomic reflections, as shown in Figure 6, which suggests a change of the propagation vector of the magnetic structure. These reflections were able to be indexed with a propagation vector **k** ≈ (0 0 0.5) with the *P*6_3_/*mmc* atomic structure with unit cell parameters *a* = 9.5562(2) Å and *c* = 7.7159(2) Å. All magnetic structures proposed by the symmetry analysis (BCS) and IsoDistort were tested, but the best result was found using a simple helical structure with the magnetic moments rotating in the *a*–*b* plane along the *c*-axis. The 4*f* and 12*k* Mn sites were split by the lowering of the magnetic symmetry into two independent orbits with a difference in rotation phases of about 120° (see the red and green arrows in Figure 8). The propagation vector was refined to the final value of **k** = (0 0 0.0483(2)).

In the previous study of the system, an easy-cone structure was suggested,^3^ which would require an angle relative to the *c*-axis to be present. While a possibility, the magnetization results presented above indicated values of around 1 mμ_B_, and they are therefore taken to be negligible. With no net moment along the *c*-axis, the structure is thus presented as a simple helical structure, visualized in Figure 8. Model comparison to data is shown in Figure SI3.

### Theoretical Calculations of Magnetic Moments

Theoretically estimated site and element-resolved magnetic moments are listed in Table 2 together with the measured data. Site-resolved magnetic moments for sites occupied by two magnetic elements within the superstructure are defined as the weighted average of the element-resolved local moments (*m*_*i*_) for a given site, that is, *m*_site_ = *∑*_*i*_*c*_*i*_*m*_*i*_, where *c*_*i*_ is given by the occupancy of each position listed in Table 1.

**Table 2:** Theoretical Site-Resolved Magnetic Moments of Mn(Co_0.78_,Ge_0.22_)_2_ in Units of μ_B_ for 0 K Collinear FM Structure together with Local Element and Site-Resolved Moments at Temperatures Well above *T*_C_ (DLM) and for Temperatures Close to *T*_C_ (LSF)^a^

atom	Wyckoff position	FM@0 K	DLM@0 K	LSF@300 K	measured@1.8 K	measured@200 K	same Mn@200 K
Mn1	4*f*	3.28	3.06	3.02	4.48(7)	2.45(5)	1.79(3)
Mn2	12*k*	3.29	3.06	3.02	3.06(3)	1.51(3)	1.79(3)
Co1	2*a*	1.70	0.68	1.05	0.63(46)	1.10(37)	1.10
Co2	6*g*	1.55	0.10	0.83	1.48(5)	0.72(5)	0.63(4)
Co3	6*h*(1)	1.73	0.42	0.94	1.65(3)	0.98(3)	1.07(3)
Co4	6*h*(2)	1.39	0.00	0.70	1.48(5)	0.72(3)	0.63(4)
Co5	12*j*	1.64	0.71	0.99	1.65(3)	0.98(3)	1.07(3)

a The moments extracted from model refinements of NPD data are also presented.

The 0 K FM data is calculated assuming a collinear FM ground state. The total magnetic moment per formula unit is 11.8 μ_B_, and it is considerably higher than the magnetometry data. Comparing the site-resolved theoretical moments to the experimental values estimated from the neutron diffraction done on 1.8 K, it can be seen that the calculated Mn moments on 4*f* and 12*k* sites are identical in contrast to the NPD-estimated local Mn moments. Also, *m*_Mn_ on 4*f* is considerably underestimated, while on 12*k*, it is slightly larger than from the magnetic structure refinements. Theoretical Co moments on 2*a*, 6*g*, and 6*h*(1) are larger, while those on 6*h*(2) and 12*j* are in satisfactory/good agreement with the experimental values measured at 1.8 K. This discrepancy comes from the assumption of a collinear FM structure while refinements show an incommensurate structure at low temperatures. On the other hand, comparing FM *m*_*i*_ values to the data obtained from the 200 K diffraction pattern, it is clear that all element-resolved local moments are considerably overestimated.

The DLM data is calculated at 0 K, but it is regarded as expected values for the DLMs at temperatures well above the Curie temperature in the paramagnetic phase.^17^ Large changes in the Co local moments (*m*_Co_) can be observed in the DLM phase compared to the FM phase. For instance, *m*_Co1_, *m*_Co3_, and *m*_Co5_ decrease considerably, while *m*_Co2_ and *m*_Co4_ almost or entirely vanish. The opposite happens with the Mn moments; they are almost unchanged. This large difference for *m*_Co_ in the FM and DLM phases indicates that thermally induced LSFs are important to take into account when magnetic properties of Mn(Co_0.78_,Ge_0.22_)_2_ are estimated from DFT. This factor may explain why both FM and DLM moments compare badly to the experimental values.

Recent theoretical investigations^25^ on the magnetic properties of Co_3.39_Mn_2_Ge_0.61_ published in ref (3) show that an excellent estimate of the Curie temperature may be achieved theoretically when thermally induced LSF of the local moments, and consequently on the magnetic exchange interactions, are taken into account. Here, the methods presented in ref (25) are used to evaluate the local moments at 300 K, a temperature close to *T*_C_. The obtained values are listed in Table 2.

It is observed that the magnitude of the Co local moments estimated at 300 K are in good agreement with those obtained from the refinements done on the 200 K NPD data, though the discrepancy regarding Mn moments is not fully resolved. Model refinements of the NPD data were performed constraining the Mn moments to having similar moments (as predicted from theoretical calculations), and this resulted in almost equally good description of the data (*R*_Bragg_ Mn-individual: 2.92; *R*_Bragg_ Mn-bound: 4.02). However, this model fails to capture some symmetry aspects, motivating the choice of model presented here. Comparing the Co and Mn moments obtained from this refinement to the LSF values, a good agreement can be seen with the Co moments, but there is still a large disagreement for the Mn moments. Refinements of the NPD data using this model are shown in the Supporting Information as Figure SI3. A model implementing the theoretically predicted Mn moments for the 1.8 K data was also constructed and is shown in Figure SI5.

**Figure 9:**
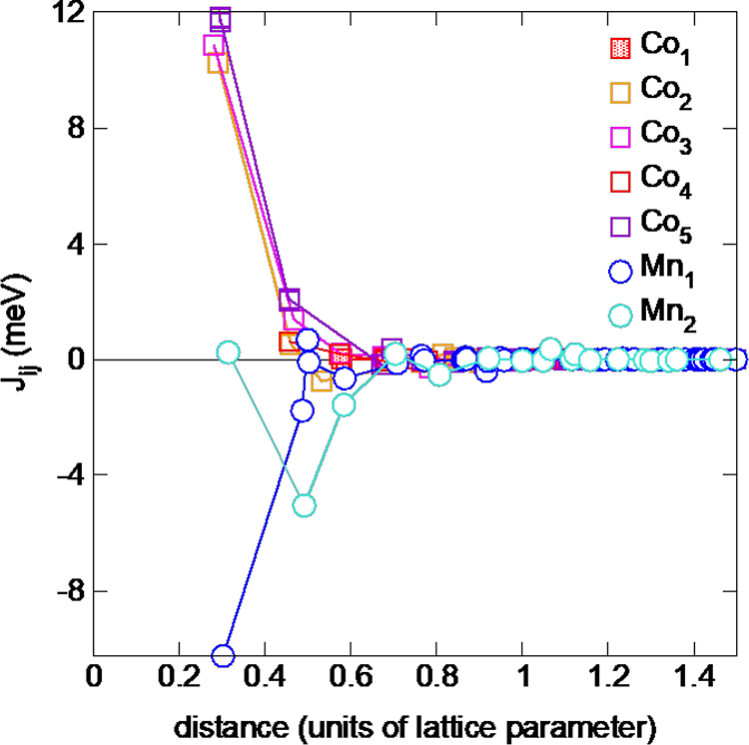
Calculated exchange parameters for Mn(Co_0.78_,Ge_0.22_)_2_ obtained from SPRKKR/CPA. Positive (negative) *J*_*ij*_ values denote FM(AF) coupling. Here, the *J*_*ij*_ values are shown for Mn_1_ (4*f* site) being at the center. Note, the Mn_2_–Mn_2_ interaction is not shown here, but the same behavior as the Mn_1_–Mn_1_ coupling is shown.

To get more insights into the system, additional calculations were performed using the fully relativistic SPRKKR starting from a FM configuration similar to the previous EMTO calculations. Limiting the calculation to a 48-atom supercell does not allow an exact reproduction of the experimental state with a long-range magnetic modulation but can reveal some basic features. The Co moments show trends similar to the experiment. The moment on the 2*a* site is strongly quenched (0.16 μ_B_), while the others range between 0.83 and 1.54 μ_B_. The moments on 6*h*(1) and 12*j* are almost identical (1.52 μ_B_ and 1.54 μ_B_), while the moments of the Co atoms on 6*g* and 6*h*(2) differ (1.37 and 0.83 μ_B_) in contrast to the experiment most likely due to the above-mentioned restrictions of the calculations. The Mn moments turn out to be similar in size, for example, being 3.30 μ_B_ (4*f*) and 3.39 μ_B_ (12*k*). The large difference derived from experimental observations could not be verified within this cell size. The *J*_*ij*_ parameters calculated based on this FM configuration point clearly to a non-collinear ground state. Strong AF couplings are observed between the Mn atoms in both inter- and intra-sublattice interactions. The AF couplings are of similar order of magnitude as the FM Mn–Co interactions, see Figure 9. The Co–Co interactions (not shown) are FM despite very small AF contributions from the next nearest-neighbor shell. As can be expected from the different magnetic moments, the *J*_*ij*_ values for couplings between Co atoms of different type show a large variation. The leading coupling parameter (nearest neighbor) is largest between Co(6*h*(1)) sites (26.5 meV) and almost vanishes for Co (2*a*). Both the AF coupling between the Mn atoms and the large spread in Co moments support the experimental conclusions of a more complex magnetic order with modulations being far more long-ranged than the 2 × 2 (in-plane) supercell can describe.

## Discussion

Ordering in hexagonal ternary Laves phases has been reported in several cases, ranging from the fully ordered Mg_2_Cu_3_Si^36^ to more complex systems such as the A′ phase (NbAlIr),^37^ Yb_6_Ir_5_Ga_7_,^38^ or Ti_36_Pt_25_Al_39_.^39^ The first report on Mn_2_Co_3_Ge could not reject either of the ordered and disordered models of the system and reported both.^4^ Similar observations have been made in other systems, as can be seen with V(Co_1–*x*_Si_*x*_)_2_,^40^ which was shown to have additional ordering for *x* = 0.43 and *x* = 0.56.^41^ The origin of the V(Co_1–*x*_Si_*x*_)_2_ superstructure was attributed to the avoidance of homo-atomic bonds,^41^ which was also proposed for Y_2_Rh_3_Ge.^42^ It is likely that a similar effect can be seen for the system presented here. The subtle reflections seen for the SCXRD patterns might have motivated the ordering discussion in the original synthesis of the system.^4^ This ordering also likely explains the discrepancy seen for the calculated structure in the recent study of the system.^3^ It should be noted that the rather similar Laves systems of Mn–Cu–Si and Mn–Ni–Si, measured by room temperature NPD, did not show any indication of additional crystallographic ordering, instead maintaining the smaller MgZn_2_-type structures.^43^

The moments of Co and Mn were predicted to achieve values of 1.56–1.59 and 3.28 μ_B_, respectively. Neither element reaches these values for the FM state. The calculations done previously assumed a temperature of *T* = 0 K, which explains the discrepancy. The Co moments recovered from the model of the incommensurate structure at 1.8 K are, however, very close to the calculated ones in the previous study.^3^ At 200 K, the magnetic moments of Co are similar to the 0.75 μ_B_ reported for the cubic Heusler phase of Co_2_MnGe,^44^ likely making the discrepancy an effect of temperature in the calculations. This is especially clear when the temperature is accounted for, as can be seen for the calculations done here.

The Mn moments present a more complicated case. Even when temperature is accounted for, the moments are lower than expected. In the systems of Mn–Cu–Si and Mn–Ni–Si,^43^ the Mn moments were reported to have values of 2.7 and 2.9 μ_B_ at room temperature, which despite ordering as AF structures was closer to the values predicted for Mn(Co_0.78_,Ge_0.22_)_2_. Furthermore, there is a pronounced difference in the size of the moment of the two Mn sites. This difference has been observed before in Mn_5_Ge_3_, with reported moments of 1.96(3) and 3.23(2) μ_B_ at 60 K for the two Mn sites, the difference of which was attributed to Mn–Mn distances rather than Ge coordination.^45^ While there is a difference in distance between Mn sites observed in Mn(Co_0.78_,Ge_0.22_)_2_, it is small compared to the difference in Mn_5_Ge_3_. It is possible that Mn on the 6*h* positions could impact Mn interactions, as calculations have shown that even small amounts of Mn on Co sites can influence the magnetic structure and Curie temperature of a system.^46^ Introduction of additional Mn on the 6*h* sites could in this case potentially allow tuning of the transition temperatures, as something similar has been observed in Mn_1.9–*x*_Co_*x*_Ge.^47^ This could make Mn(Co_0.78_,Ge_0.22_)_2_ an interesting candidate for a magnetocaloric material.

The incommensurate structure has pairs of Mn moments propagating through the system. The angle between these pairs vary but has a value close to 120°, something commonly observed in frustrated magnetic systems.^48^ Despite this, AC magnetometry measurements gave no indication of magnetic frustration, suggesting that more complicated interactions are the reason for this particular alignment of Mn moments. It should be noted that the Mn moment on the 4*f* position is very large. Other Mn-containing systems have reported similar or even larger values such as Mn_3_As_2_,^49^ where the Mn moment was reported to be 4.48 μ_B_, while in Pd_3_Mn,^50^ a value as large as 5.2 μ_B_ was seen.

## Conclusions

The atomic and magnetic structures of Mn(Co_0.78_,Ge_0.22_)_2_ have been determined using first-principles calculations, magnetometry, and neutron and X-ray diffraction. The system was shown to form a superstructure described by a unit cell twice the length in the *a*- and *b*-directions in the space group *P*6_3_/*mmc*. Below 329 K, the compound ordered ferromagnetically in the magnetic space group *P*6_3_/*mm*′*c*′. Further decrease of the temperature past *T* < 168 K resulted in the system adopting an incommensurate structure with a propagation vector **k** = (0 0 0.0483), which was determined to be a helical spin structure.

This study highlights how the careful evaluation of theoretical results can lead to surprising findings. While originally envisioned as a permanent magnet, Mn(Co_0.78_,Ge_0.22_)_2_ provides the possibility of creating a tunable magnetocaloric material. In addition to providing a new magnetic system with potential applications, it also provides a new testbed for theoretical understanding of Mn–Mn interactions.

## Supporting Information Available

Plots of Rietveld model refinements and values extracted from them (PDF)
