# Supplementary material for: Revealing the Magnetic Structure and Properties of Mn(Co,Ge)2
Source: Inorg Chem. 2022 Oct 21;61(44):17673–81. doi: 10.1021/acs.inorgchem.2c02758 (PMC9644371; doi:10.1021/acs.inorgchem.2c02758)
Supplement: Supplementary file 1 — ic2c02758_si_001.pdf [file ic2c02758_si_001.pdf]

Revealing the Magnetic Structure and Properties of $Mn(Co,Ge)_2$

Simon R. Larsen*,[*,†] Vitalii Shtender,[†] Daniel Hedlund,[‡] Erna K. Delczeg-Czirjak,[¶] Premysl Beran,[§,#] Johan Cedervall,[‖] Alena Vishina,[¶] Thomas C. Hansen,[⊥] Heike C. Herper,[¶] Peter Svedlindh,[‡] Olle Eriksson,[¶,@] and Martin Sahlberg[†]

†*Department of Chemistry - Ångström, Uppsala University, Box 538, 751 21, Uppsala, Sweden*

‡*Department of Materials Science and Engineering, Uppsala University, Box 35, 751 03 Uppsala, Sweden*

¶*Department of Physics and Astronomy, Uppsala University, Box 516, SE-75120, Uppsala, Sweden*

§*European Spallation Source ESS ERIC, Box 176, 221 00, Lund, Sweden*

‖*Department of Materials and Environmental Chemistry, Stockholm University, 10691 Stockholm, Sweden*

⊥*Institut Laue-Langevin, 71 avenue des Martyrs, 38000 Grenoble, France*

#*Nuclear Physics Institute, ASCR, Hlavni 130, 25068 Rez, Czech Republic*

@*School of Science and Technology, Örebro University, SE-701 82 Örebro, Sweden*

E-mail: simon.rosenqvist.larsen@kemi.uu.se

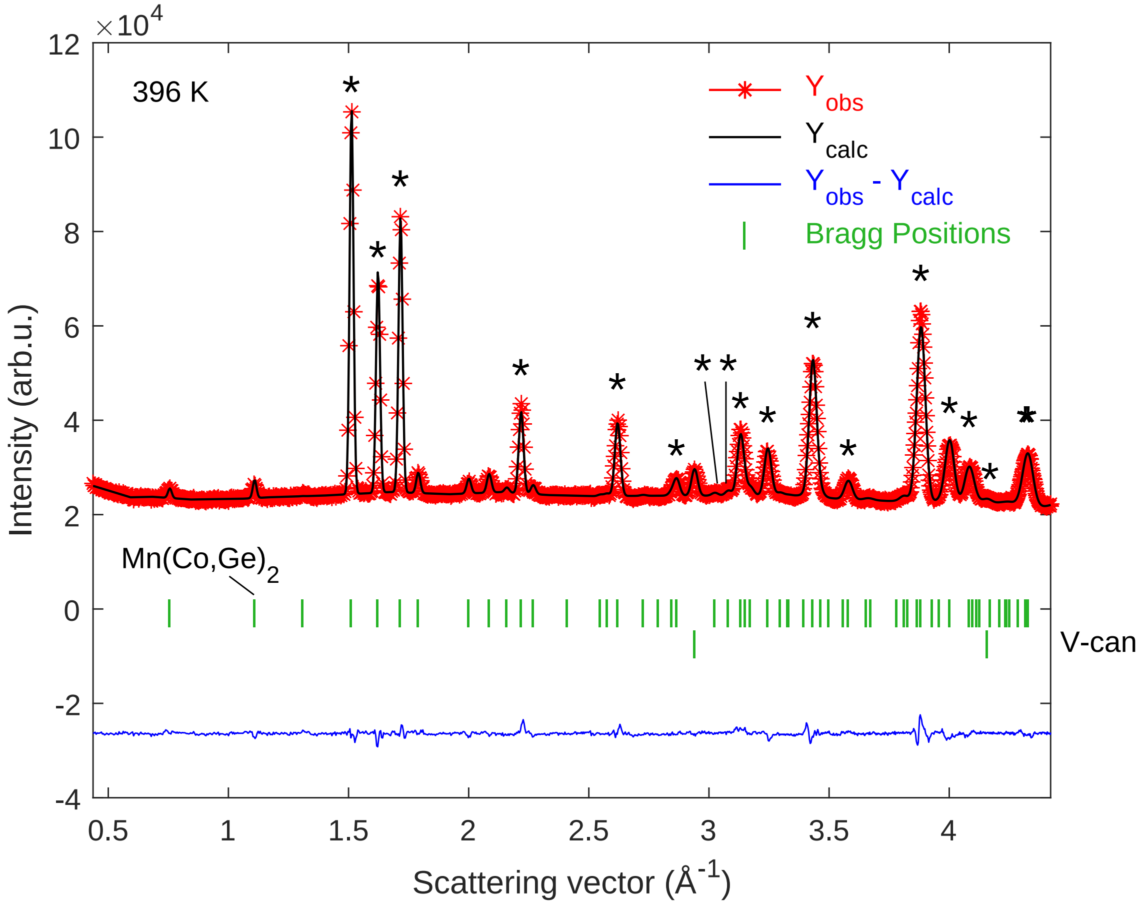

Figure SI1: Comparison of the measured and calculated diffraction patterns of $Mn(Co_{0.78},Ge_{0.22})_2$ in the paramagnetic state at 396 K. The black asterisks denote the reflections of the $MgZn_2$-type unit cell. $R_p = 1.13$, $R_{wp} = 1.59$, $R_{exp} = 0.25$.

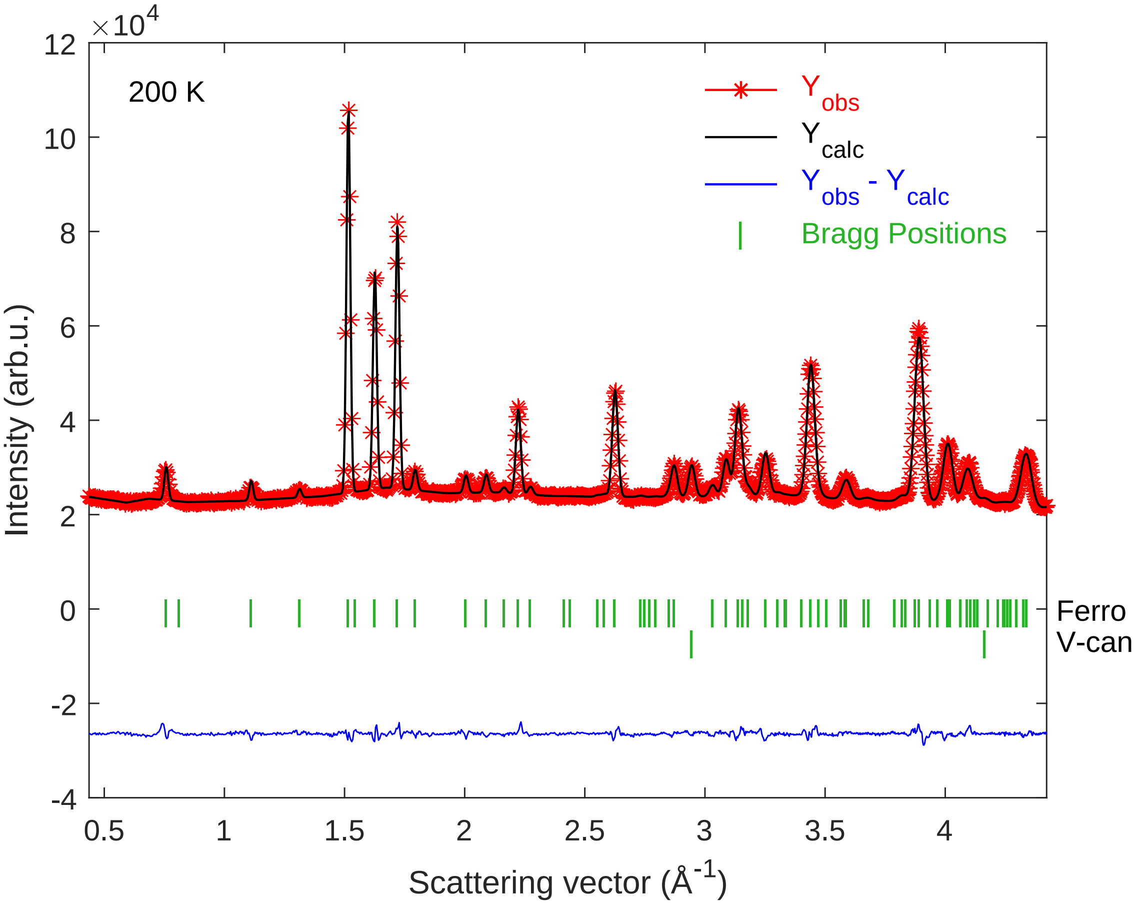

Figure SI2: Comparison of the refined model of the ferromagnetic structure of $Mn(Co_{0.78},Ge_{0.22})_2$ and the data at 200 K. The system assumes the magnetic space group $P6_3/mm'c'$. $R_p = 1.16$, $R_{wp} = 1.59$, $R_{exp} = 0.25$.

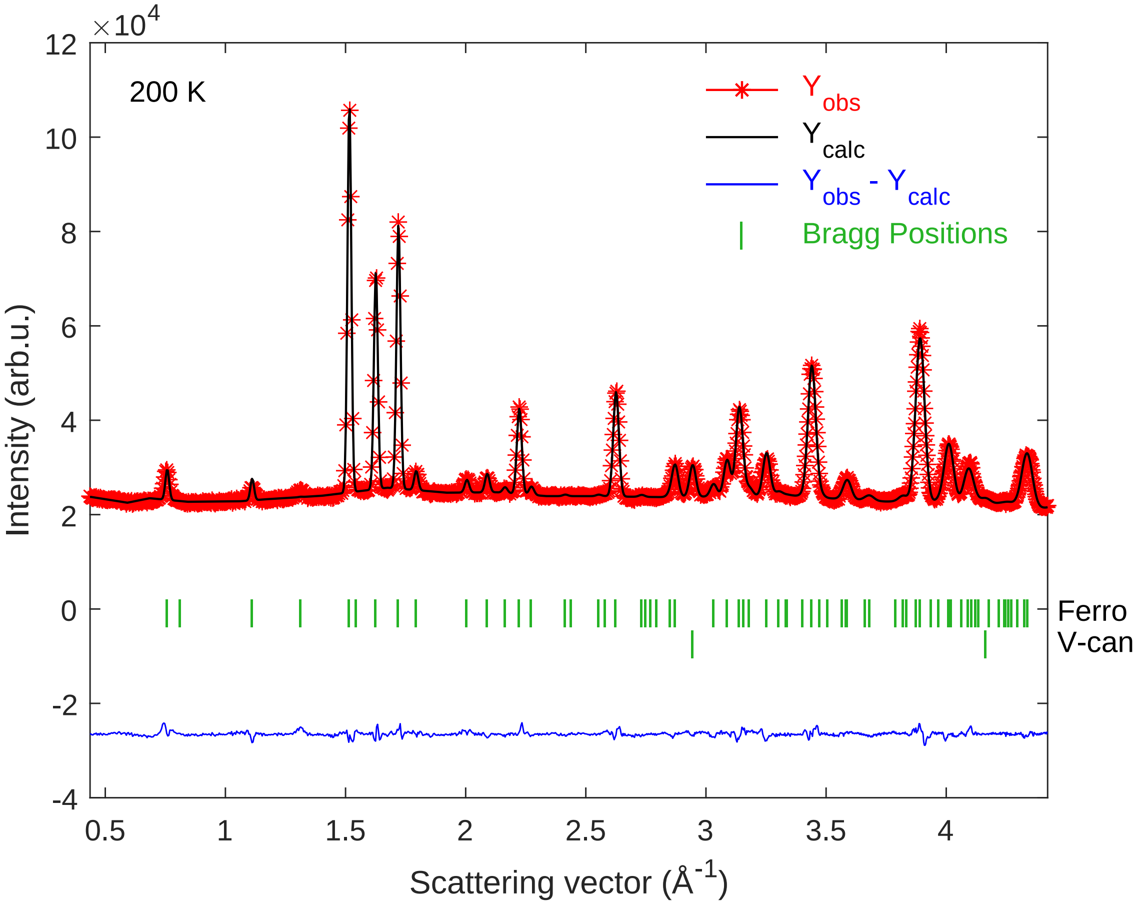

Figure SI3: Model refinement of the magnetic structure of $Mn(Co_{0.78},Ge_{0.22})_2$ at 200 K with the Mn moments constrained to having similar values. Note the lack of intensity for the reflection at 1.3 Å^{-1}. $R_p = 1.25$, $R_{wp} = 1.70$, $R_{exp} = 0.25$.

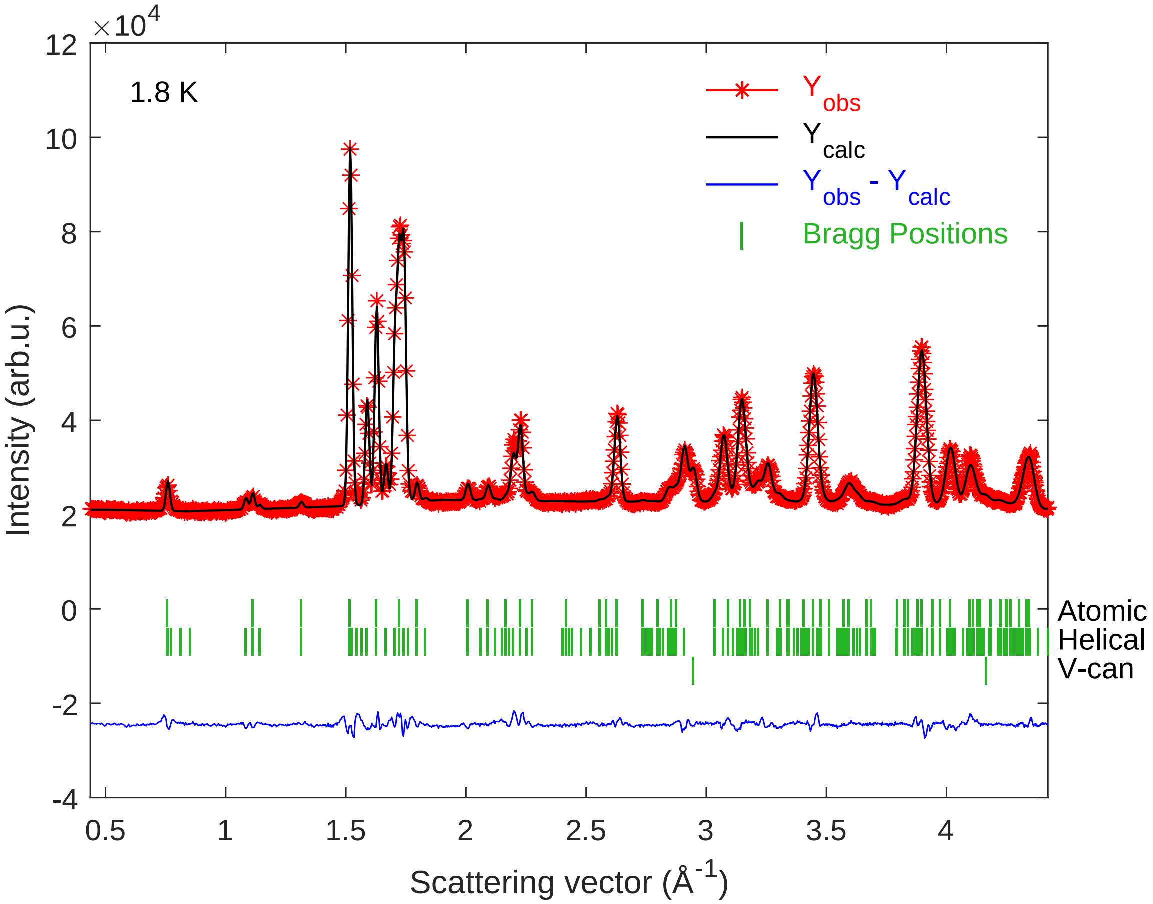

Figure SI4: The refined model of the incommensurate magnetic structure of $Mn(Co_{0.78},Ge_{0.22})_2$ compared to the diffraction data measured at 1.8 K. $R_p = 1.16$, $R_{wp} = 2.11$, $R_{exp} = 0.25$.

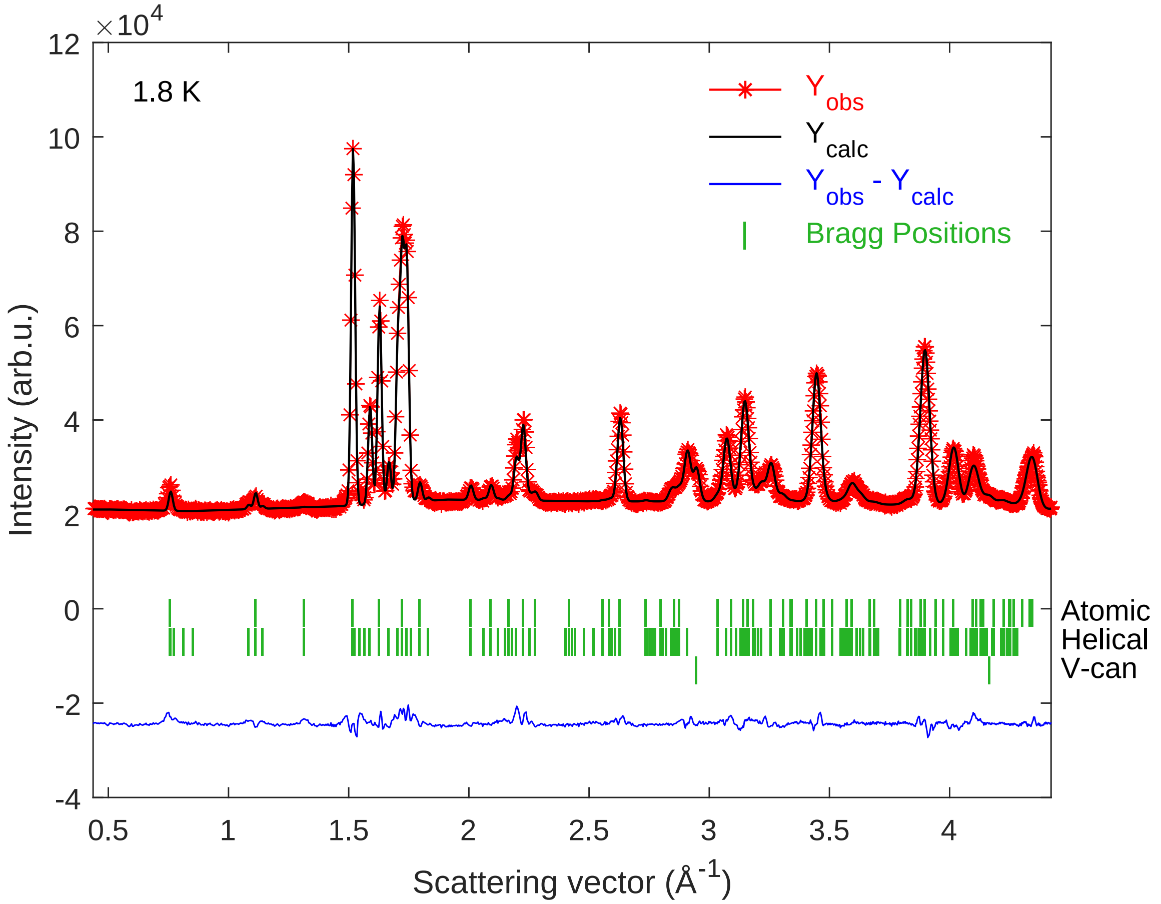

Figure SI5: The refined model of the magnetic structure of $Mn(Co_{0.78},Ge_{0.22})_2$ at 1.8 K using theoretically calculated Mn moments compared to the diffraction data. The Co moments were fixed to the values extracted from the refinement seen in Figure SI4. Note the lack of intensity for the reflection at 1.3 $Å^{-1}$. $R_p = 1.69$, $R_{wp} = 2.35$, $R_{exp} = 0.25$.

Table SI1: Atomic positions and related magnetic moments extracted from the refined model of the ferromagnetic system of Mn($Co_{0.78}$,$Ge_{0.22}$)$_2$ at 200 K together with predicted theoretical values (LSF) when thermally induced longitudinal spin fluctuations are taken into account.

Atom	Wyckoff position	x	y	z	Moment (μ_B)	Predicted
Mn1	$4f$	1/3	2/3	0.0575(15)	2.45(5)	3.02
Mn2	$12k$	0.1672(7)	0.3343(47)	0.5675(5)	1.51(3)	3.02
Co1	$2a$	0	0	0	1.10(37)	1.05
Co2	$6g$	0.5	0	0	0.72(5)	0.83
Co3	$6h(1)$	0.5849(18)	0.1698(37)	0.25	0.98(3)	0.94
Co4	$6h(2)$	0.0857(5)	0.1713(9)	0.25	0.72(3)	0.70
Co5	$12j$	0.0864(9)	0.4149(9)	0.25	0.98(3)	0.99

Table SI2: The atomic positions and magnetic moments extracted from model refinements of the data measured at 1.8 K of Mn($Co_{0.78}$,$Ge_{0.22}$)$_2$ together with theoretically predicted values for the 0 K collinear ferromagnetic (FM) structure.

Atom	Wyckoff position	x	y	z	Moment (μ_B)	Predicted FM
Mn1	$4f$	1/3	2/3	0.0549(4)	4.48(7)	3.28
Mn2	$12k$	0.1689(3)	0.3357(5)	0.5666(1)	3.06(3)	3.29
Co1	$2a$	0	0	0	0.63(46)	1.70
Co2	$6g$	0.5	0	0	1.48(5)	1.55
Co3	$6h(1)$	0.5878(4)	0.1756(8)	0.25	1.65(3)	1.73
Co4	$6h(2)$	0.0860(4)	0.1721(9)	0.25	1.48(5)	1.39
Co5	$12j$	0.0852	0.4142	0.25	1.65(3)	1.64
